# Hybrid Biomaterial Initiates Refractory Wound Healing via Inducing Transiently Heightened Inflammatory Responses

**DOI:** 10.1002/advs.202105650

**Published:** 2022-05-23

**Authors:** Xuemei Liu, Geng Dou, Zihan Li, Xiangdong Wang, Ronghua Jin, Yao Liu, Huijuan Kuang, Xiaoyao Huang, Xiaoxue Yang, Xiaoshan Yang, Siying Liu, Meiling Wu, Hao Guo, Feng Ding, Haokun Xu, Shiyu Liu, Yan Jin, Kun Xuan

**Affiliations:** ^1^ State Key Laboratory of Military Stomatology and National Clinical Research Center for Oral Diseases and Shaanxi Clinical Research Center for Oral Diseases Department of Preventive Dentistry School of Stomatology The Fourth Military Medical University Xi'an 710032 China; ^2^ State Key Laboratory of Military Stomatology and National Clinical Research Center for Oral Diseases and Shaanxi International Joint Research Center for Oral Diseases Center for Tissue Engineering School of Stomatology The Fourth Military Medical University Xi'an 710032 China; ^3^ Department of Pediatric Dentistry School and Hospital of Stomatology China Medical University Liaoning Provincial Key Laboratory of Oral Diseases Shenyang 110002 China; ^4^ Guangxi Key Laboratory of Bioactive Molecules Research and Evaluation and College of Pharmacy Guangxi Medical University Nanning 530021 China; ^5^ Stomatology Hospital Southern Medical University Guangzhou 510280 China

**Keywords:** biomaterials, inflammation, macrophages, neutrophils, refractory wounds

## Abstract

Inflammation plays a crucial role in triggering regeneration, while inadequate or chronic inflammation hinders the regenerative process, resulting in refractory wounds. Inspired by the ideal regeneration mode in lower vertebrates and the human oral mucosa, realigning dysregulated inflammation to a heightened and acute response provides a promising option for refractory wound therapy. Neutrophils play important roles in inflammation initiation and resolution. Here, a hybrid biomaterial is used to stimulate transiently heightened inflammatory responses by precise tempospatial regulation of neutrophil recruitment and apoptosis. The hybrid biomaterial (Gel@fMLP/SiO_2_‐FasL) is constructed by loading of formyl‐met‐leu‐phe (fMLP) and FasL‐conjugated silica nanoparticles (SiO_2_‐FasL) into a pH‐responsive hydrogel matrix. This composition enables burst release of fMLP to rapidly recruit neutrophils for heightened inflammation initiation. After neutrophils act to produce acids, the pH‐responsive hydrogel degrades to expose SiO_2_‐FasL, which induces activated neutrophils apoptosis via FasL‐Fas signaling triggering timely inflammation resolution. Apoptotic neutrophils are subsequently cleared by macrophages, and this efferocytosis activates key signalings to promote macrophage anti‐inflammatory phenotypic transformation to drive regeneration. Ultimately, Gel@fMLP/SiO_2_‐FasL successfully promotes tissue regeneration by manipulating inflammation in critical‐sized calvarial bone defects and diabetic cutaneous wound models. This work provides a new strategy for refractory wound therapy via inducing transiently heightened inflammatory responses.

## Graphical abstract

Inflammation plays a vital role in tissue regeneration, while an insufficient and prolonged inflammatory process will result in refractory wounds. A hybrid biomaterial is constructed here to manipulate inflammation to a transiently heightened process by precise tempospatial regulation of neutrophil recruitment and apoptosis, providing a new strategy for refractory wound therapy.

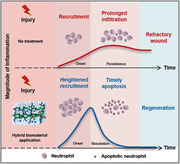

## 1 Introduction

Favorable regeneration of damaged tissues would bring tremendous benefits for addressing a number of medical challenges and social burdens. Regeneration capacity in mammals is very limited; however, certain lower vertebrates, such as zebrafish, exhibit near‐perfect regeneration in a broad spectrum of tissues that is not achieved in mammals.^[^ ^1^ ^]^ Thus, understanding regeneration mechanisms in highly regenerative species would provide instructive clues for the development of regenerative strategies for mammalian tissues. Evidence has suggested that an acute and transient inflammatory response after injury is essential for efficient regeneration in highly regenerative animals.^[^ ^2^, ^3^ ^]^ Moreover, the accelerated scarless healing in the human oral mucosa is also closely linked to a heightened inflammatory response that is primed at baseline and the rapid resolution of inflammation compared to that in skin.^[^ ^4^ ^]^ In contrast, an ineffective inflammatory response characterized by decreased neutrophil and macrophage recruitment or relatively chronic inflammation that manifests as persist increased immune cell infiltration and proinflammatory cytokines expression will result in refractory wounds characterized by chronic healing or even nonhealing. Refractory wounds, such as critical‐sized bone defects and diabetic skin ulcers, can further lead to infection, invalidity and even disability.^[^ ^5^, ^6^, ^7^ ^]^ Therefore, strategies to elicit an effectively heightened and transiently acute inflammatory response can facilitate tissue regeneration and refractory wound healing.

Inflammation is a tightly regulated process with the aim of eliminating pathogenic insult and removing damaged tissue to restore tissue homeostasis.^[^ ^8^, ^9^ ^]^ After injury, neutrophils, monocytes and macrophages are major immune cells that cooperate during the onset, progression and resolution of inflammation.^[^ ^10^ ^]^ Neutrophils are typically the first leukocytes that are recruited to sites of inflammation and are capable of eradicating pathogens and clearing debris.^[^ ^11^ ^]^ In addition, accumulating evidence suggests that neutrophils contribute to tissue repair by releasing a variety of mediators, such as growth factors, proangiogenic factors and microvesicles.^[^ ^12^ ^]^ However, excessive infiltration or persistence of neutrophils results in collateral tissue damage, chronic inflammation and impaired tissue regeneration.^[^ ^13^ ^]^ Therefore, once their work is done, neutrophils must be removed from the site of injury. Concomitantly, neutrophil clearance is essential for initiating the resolution phase of inflammation and ensuring the safe conclusion of the inflammatory response.^[^ ^14^, ^15^ ^]^ The clearance of neutrophils generally occurs by apoptosis and subsequent engulfment by macrophages (efferocytosis). Efferocytosis leads to macrophage reprogramming from a proinflammatory to an anti‐inflammatory phenotype, which contributes to the restoration of homeostasis and promotes regeneration.^[^ ^16^, ^17^, ^18^ ^]^ Therefore, triggering the rapid recruitment of neutrophils and inducing their apoptosis in a timely manner would induce transiently heightened inflammation primers for regeneration; however, specific manipulation strategies are not yet available.

**Figure 1:**
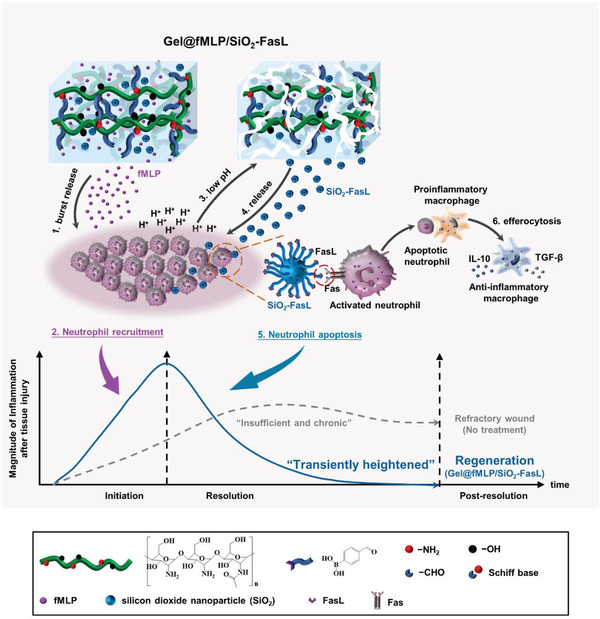
Schematic illustration of the Gel@fMLP/SiO_2_‐FasL for transiently heightened inflammatory response manipulation to initiate refractory wound healing. SiO_2_‐FasL, silicon dioxide nanoparticles conjugated with FasL; Gel@fMLP/SiO_2_‐FasL, pH‐responsive hydrogel matrix loaded with fMLP and SiO_2_‐FasL.

In this study, a hybrid biomaterial was constructed to reprogram the inflammatory process. Formyl‐met‐leu‐phe (fMLP) is the most effective chemoattractant for neutrophils.^[^ ^19^, ^20^, ^21^ ^]^ Fas ligand (aggregated or membrane‐bound) serves as a potent trigger for apoptosis by interacting with Fas, which is expressed on activated neutrophils.^[^ ^22^, ^23^ ^]^ fMLP and FasL‐conjugated silica dioxide nanoparticles (SiO_2_‐FasL) were loaded in a pH‐responsive hydrogel system (Gel@fMLP/SiO_2_‐FasL) to exert precise tempospatial regulation of neutrophil infiltration and fate for refractory wound healing. Specifically, Gel@fMLP/SiO_2_‐FasL enabled the burst release of fMLP to recruit neutrophils rapidly for heightened inflammation initiation, followed by timely SiO_2_‐FasL induced neutrophil apoptosis and subsequent macrophage transformation to an anti‐inflammatory phenotype for inflammation resolution, which reestablished a transiently heightened inflammatory response (**Figure** **1**) and contributed to critical‐sized calvarial bone defect repair and diabetic cutaneous wound healing.

## 2 Results

### 2.1 Preparation and Characterization of the Hybrid Biomaterial (Gel@fMLP/SiO_2_‐FasL)

To transiently heighten the inflammatory response in refractory wounds, we designed a phenylboronic acid (PBA)‐based polymeric hydrogel loaded with fMLP/SiO_2_‐FasL complexes (Gel@fMLP/SiO_2_‐FasL). The hybrid biomaterial was constructed to precisely regulate neutrophil recruitment and apoptosis in a two‐stage manner for inflammation initiation and resolution. The first stage involved the release of fMLP, the most effective neutrophil chemoattractant, into the external environment through quick diffusion. The second stage involved pH‐triggered delivery of SiO_2_‐FasL, a potent trigger of apoptosis in activated neutrophils via FasL‐Fas signaling, from the hydrogel matrix to the acidic microenvironment.

**Figure 2:**
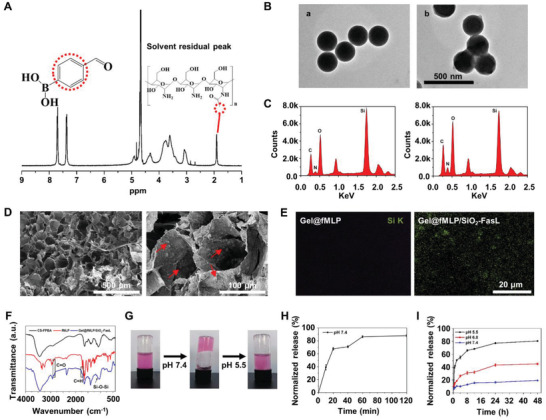
Preparation and characterization of Gel@fMLP/SiO_2_‐FasL. A) The 1H NMR spectrum of FPBA functionalized CS. B) TEM images of SiO_2_ a) before and b) after FasL immobilization. Scale bars, 500 nm. C) EDS of SiO_2_ before and after FasL immobilization. D) SEM image of the colloidal hydrogel (Gel@fMLP/SiO_2_‐FasL). E) EDS analysis of Gel@fMLP and Gel@fMLP/SiO_2_‐FasL containing the mapping analysis of Si. F) IR spectrometry of CS‐FPBA, fMLP and Gel@fMLP/SiO_2_‐FasL. G) Photographs of the gelation process of the Gel@fMLP/SiO_2_‐FasL with a solution containing RhB (representing fMLP) and SiO_2_‐FasL after incubation at pH 7.4. The decomposition of Gel@fMLP/SiO_2_‐FasL at pH 5.5. H) The in vitro release profile of fMLP from Gel@fMLP/SiO_2_‐FasL. I) The in vitro pH‐dependent release profile of SiO_2_‐FasL from Gel@fMLP/SiO_2_‐FasL.

The PBA‐based polymeric hydrogels were formed using chitosan (CS) modified by 4‐formylphenylboronic acid (FPBA) as described previously.^[^ ^24^ ^]^ After the sample was purified, the 1H nuclear magnetic resonance (1H NMR) spectrum showed the characteristic resonance signals of FPBA, such as phenyl protons at 7.36–7.71 ppm, which confirmed the formation of CS‐FPBA (**Figure** **2**A). Meanwhile, the peak intensities of the hydrogen protons on the phenyl ring of FPBA and the methyl protons of CS at ≈1.9 ppm demonstrated the conjugated FPBA content in CS‐FPBA (Figure 2A). The strengthening of characteristic vibration of C═N (1665 cm^−1^) and C—N (1370 cm^−1^) indicated that Schiff base was formed between the NH_2_ group from CS and the aldehyde (CHO) group from FPBA (Figure S1A, Supporting Information). Moreover, CS had no UV absorption peaks between 220 and 400 nm; however, a strong FPBA absorption peaked at ≈270 nm in the UV spectrum of CS‐FPBA that was consistent with the absorption peaks of benzene ring in phenylboronic acid (Figure S1B, Supporting Information). These data demonstrated that FPBA is successfully conjugated to CS.

FasL was immobilized onto the surface of SiO_2_ through amidation reactions between COOH in FasL and NH_2_ on SiO_2_ to form SiO_2_‐FasL, which can induce apoptosis in activated neutrophils. FasL residing on SiO_2_ surface was observed by transmission electron microscopy (TEM) (Figure 2B). Signals corresponding to the element N in the loaded FasL (Figure 2C) were detected by energy‐dispersive X‐ray spectroscopy (EDS) analysis. Moreover, the IR spectrometry showed an obvious bovine serum albumin (BSA) (representing FasL) signal, indicating SiO_2_‐NH_2_ could be successfully modified by FasL (Figure S2A, Supporting Information). The zeta potential changes in SiO_2_‐NH_2_ and SiO_2_‐FasL from ≈−10.0 to −22.7 mV, could be ascribed to the depletion of surface amino groups (Figure S2B, Supporting Information). In addition, the size of SiO_2_‐NH_2_ was ≈200 nm, and which was increased to 220 nm after being immobilized with FasL (SiO_2_‐FasL) through 1‐ethyl‐3‐(3‐dimethylaminopropyl) carbodiimide hydrochloride (EDC)/*N*‐hydroxysuccinimide (NHS) (Figure S2C, Supporting Information). Finally, the loading percentage of FasL was measured by bicinchoninic acid (BCA) assay with BSA (representing FasL), the results showed that the loading percentage reached 40% (Figure S2D, Supporting Information). These results demonstrated that FasL was successfully immobilized on SiO_2_, forming FasL‐surface functionalized SiO_2_ (SiO_2_‐FasL).

**Figure 3:**
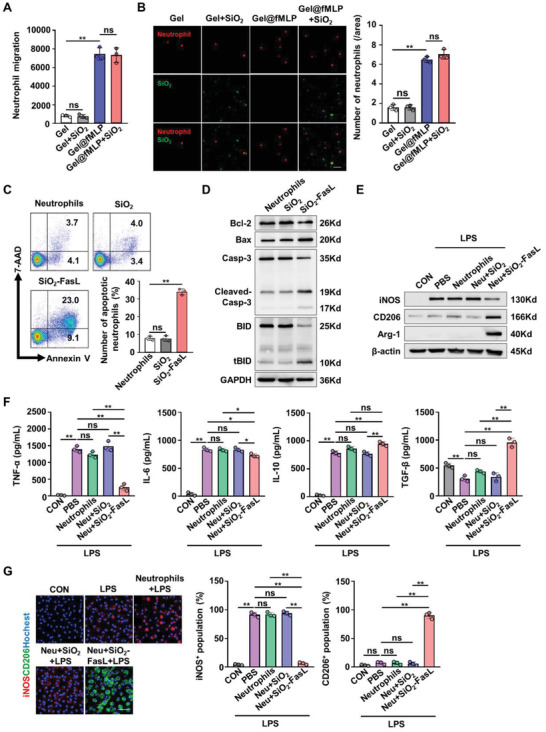
Regulation of neutrophil migration and apoptosis and macrophage polarization by the hybrid biomaterial in vitro. A) Quantitative flow cytometric analysis of neutrophil migration after the different treatments. B) Representative fluorescence images and quantitative analysis of neutrophil (red) migration to the lower chamber of transwell. Scale bars, 50 µm. C) Representative flow cytometry plots and quantitative analysis of neutrophil apoptosis. D) Western blot analysis of apoptosis‐related proteins in neutrophils. E) Western blot analysis of the phenotypic markers in LPS‐stimulated macrophages. Proinflammatory macrophage marker, iNOS; anti‐inflammatory macrophage markers, CD206 and Arg‐1. F) The levels of cytokines released by macrophages measured by enzyme‐linked immunosorbent assay (ELISA). G) Representative fluorescence images of macrophage phenotypes after incubation with different pretreated neutrophils; iNOS (red), CD206 (green), and nuclei (blue). Scale bars, 50 µm. All data were generated from at least three independent experiments and are presented as the means ± standard deviation (SD). Statistical analysis was performed by one‐way ANOVA. ns, not significant; *P < 0.05 and **P < 0.01.

The PBA‐based polymeric hydrogels were then fabricated by mixing an aqueous solution of fMLP and SiO_2_‐FasL (30 wt%) with a CS‐FPBA aqueous solution to obtain Gel@fMLP/SiO_2_‐FasL. The hydrogel matrix had a porous structure with pore sizes of ≈20 µm in diameter (Figure 2D). Scanning electron microscopy (SEM) images of the colloidal hydrogel showed numerous SiO_2_ conjugated in the hydrogel (red arrows). The presence of SiO_2_ was also confirmed by mapping, which showed an obvious elemental signal of Si (Figure 2E). Moreover, the chemical component of this hydrogel was further verified by IR spectrometry. The results showed an obvious fMLP signal, as indicated by the clear characteristic vibration of C═O (1720 cm^−1^) from —COOH groups and C—H (2860 cm^−1^), as well as the characteristic vibration of silicon from Si–O–Si (1095 cm^−1^) (Figure 2F). As shown in Figure 2G, the hydrogels can be loaded with RhB (representing fMLP) and SiO_2_‐FasL through physical adsorption, and the red dye can be observed throughout the matrix of the hydrogels. Moreover, under low pH condition (pH 5.5), significant pH‐induced dissociation of the hydrogel occurred, ensuring low pH‐mediated fast release of SiO_2_‐FasL (Figure 2G). The on‐demand release profile showed that there was quick release of ≈75% RhB from the hydrogel matrix after 60 min (Figure 2H), indicating that fMLP can be easily released from the hydrogel. In contrast, ≈15% fluorescein isothiocyanate (FITC)‐labeled SiO_2_‐BSA (representing SiO_2_‐FasL) was released from the hydrogel after 48 h at pH 7.4. However, a further decrease in the pH value to 6.8 and 5.5 could trigger cumulative release of up to 40% and 80% SiO_2_‐BSA‐FITC over 48 h, respectively, demonstrating that the pH‐responsive controlled release of SiO_2_‐FasL could be achieved (Figure 2I). Moreover, the storage modulus *G*' and the lost modulus *G*' of the hydrogel remained unchanged within a certain strain range, indicating that the hydrogel could withstand large deformation and maintain a complete 3D network structure with good stability (**Figure** **3**S, Supporting Information).

Taken together, these results indicated that Gel@fMLP/SiO_2_‐FasL could rapidly release fMLP and perform controlled release of SiO_2_‐FasL in respond to low pH, which was the basis for the tempospatial regulation of neutrophil recruitment and apoptosis to manipulate the inflammatory response for refractory wound healing.

### 2.2 The Hybrid Biomaterial Induced Neutrophil Migration and Apoptosis and Macrophage Phenotypic Transformation In Vitro

Neutrophils act as the first responders of the innate immune system after injuries or infections, and the recruitment of neutrophils represents the initiation of inflammation.^[^ ^11^ ^]^ The hybrid biomaterial was designed to rapidly recruit neutrophils to injury sites through the burst release of fMLP. To confirm the chemotactic effect, neutrophils were isolated (Figure S4A, Supporting Information) and incubated in the upper chamber of a transwell with prepared hydrogel supernatants in the lower chamber for 45 min. The number of cells that migrated to the lower chamber was recorded and calculated by flow cytometry. The results showed that hydrogels loaded with fMLP (Gel@fMLP and Gel@fMLP + SiO_2_) significantly promoted cell migration; however, the effects of hydrogel (Gel) and hydrogel loaded with SiO_2_ (Gel@SiO_2_) were negligible (**3A**). As shown by fluorescence imaging, the supernatants of Gel@fMLP supplemented with or without SiO_2_ (Gel@fMLP and Gel@fMLP + SiO_2_) induced neutrophil migration to the lower chamber, while almost no effects were observed in response to the supernatants without fMLP loading (Gel and Gel + SiO_2_) (Figure 3B). These results indicated that hybrid biomaterial loaded with fMLP could effectively recruit neutrophils in vitro.

Neutrophil apoptosis and clearance are prerequisites for successful inflammation resolution.^[^ ^14^ ^]^ In addition to intrinsic spontaneous apoptosis, neutrophils are killed by extrinsic death receptor‐induced apoptosis. The Fas/FasL pathway is a key extrinsic signal for neutrophil apoptosis.^[^ ^22^ ^]^ The silicon dioxide nanoparticles were designed conjugated with FasL (SiO_2_‐FasL) to trigger programmed cell death by binding to Fas on activated neutrophils. To verify the effects of SiO_2_‐FasL, neutrophils were first activated and the expression of myeloperoxidase (MPO) and Fas on neutrophils was shown to be elevated (Figure S4B, Supporting Information). Then, the activated neutrophils were cocultured with SiO_2_‐FasL or nanoparticles without FasL (SiO_2_) for 6 h. Compared to the control groups (SiO_2_ and untreated activated neutrophils), SiO_2_‐FasL significantly promoted apoptosis in activated neutrophils, as assessed by flow cytometry (Figure 3C). In addition, Western blot analysis showed that the expression of the apoptosis‐related proteins, B‐cell lymphoma‐2 (Bcl‐2), caspase‐3 and Bcl‐2 homology‐interacting domain death agonist (BID) was downregulated by SiO_2_‐FasL. Moreover, the expression of Bcl‐2‐associated X protein (Bax), cleaved caspase‐3, and tBID (the truncated form of BID) in neutrophils was upregulated by SiO_2_‐FasL (Figure 3D). These data suggested that apoptosis could be induced in activated neutrophils by SiO_2_ conjugated with FasL.

Apoptotic neutrophils are rapidly engulfed by macrophages through a process called efferocytosis, which promotes macrophage polarization and helps to limit inflammation. The phenotypic switch in macrophages from proinflammatory cells to anti‐inflammatory cells and the development of a remodeling phenotype is key to an efficient repair process.^[^ ^16^, ^25^ ^]^ To investigate the effect of the hybrid biomaterial on macrophage polarization after inducing neutrophil apoptosis in vitro, bone marrow‐derived macrophages (BMDMs) were first isolated (Figure S5, Supporting Information). Then, the inflammatory macrophages were stimulated by lipopolysaccharide (LPS) and subjected to different treatments, including phosphate‐buffered saline (PBS), untreated activated neutrophils, activated neutrophils cultured with SiO_2_ (Neu + SiO_2_), and activated neutrophils cultured with SiO_2_‐FasL (Neu + SiO_2_‐FasL), while unstimulated macrophages served as a control. Western blot analysis showed that Neu + SiO_2_‐FasL reduced the expression of the proinflammatory protein inducible nitric oxide synthase (iNOS), while the levels of the anti‐inflammatory proteins CD206 and arginase‐1 (Arg‐1) were enhanced (Figure 3E). No effects were observed in response to untreated activated neutrophils and Neu + SiO_2_ compared to PBS treatment. Immune factors secreted in the supernatants were then measured, and the results indicated that Neu + SiO_2_‐FasL significantly inhibited the secretion of the proinflammatory cytokines tumor necrosis factor‐*α* (TNF‐*α*) and interleukin‐6 (IL‐6). The secretion of the anti‐inflammatory factors IL‐10 and transforming growth factor‐*β* (TGF‐*β*) was enhanced by Neu + SiO_2_‐FasL (Figure 3F). Moreover, iNOS‐positive cells were increased after LPS stimulation as visualized by confocal microscopy, and were repressed by treatment with Neu + SiO_2_‐FasL. The expression of CD206 was also enhanced in the Neu + SiO_2_‐FasL group, and the phenotypic change in macrophages was not observed in the groups exposed to untreated activated neutrophils and Neu + SiO_2_ (Figure 3G). These results demonstrated that apoptotic neutrophils induced by SiO_2_‐FasL could promote macrophage polarization after being phagocytosed by macrophages.

Collectively, these findings suggested that the hybrid biomaterial could effectively recruit neutrophils through fMLP, induce apoptosis in activated neutrophils by FasL‐Fas signaling and then promote macrophage transformation to an anti‐inflammatory phenotype in vitro.

### 2.3 Gel@fMLP/SiO_2_‐FasL Improved the Repair of Critical‐Sized Calvarial Bone Defects

Large bone defects have long been recognized as a great clinical concern because they can complicate osteosynthesis and may lead to delayed healing or nonhealing. Bone healing is a multistage regenerative process, and acute inflammation is crucial in the response to injury which initiates the onset of bone repair.^[^ ^26^, ^27^ ^]^ However, insufficient or prolonged inflammation can impair the healing process.^[^ ^28^ ^]^ Therefore, a transiently heightened inflammatory response that promotes endogenous bone regeneration provides us with a new direction.

**Figure 4:**
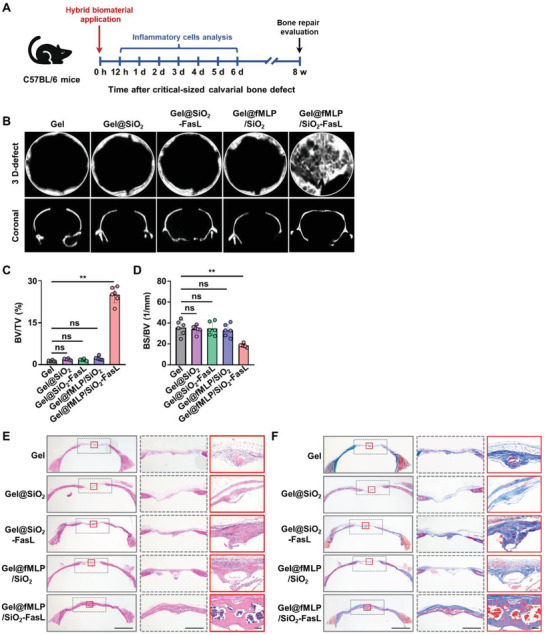
Gel@fMLP/SiO_2_‐FasL improves the repair of critical‐sized calvarial bone defects in vivo. A) Critical‐sized calvarial bone defects were generated in 8 week old female C57BL/6 mice and treated with hydrogels hybridized with different components. The mice were sacrificed at different time points post operation for further examination. B) Micro‐CT analysis of bone defects. C,D) Quantitative analysis of BV/TV and BS/BV. E) Representative images showing hematoxylin and eosin (H&E) staining of the calvarial bone defects. Scale bars, 1 mm (left), 2 mm (middle), 75 µm (right). F) Representative images of Masson staining showing the calvarial bone defects. Scale bars, 1 mm (left), 2 mm (middle), 75 µm (right). *n* = 6 per group. The data are presented as the means ± SD. In (C), statistical analysis was performed using the Kruskal–Wallis *H*‐test. In (D), statistical analysis was performed using one‐way ANOVA. ns, not significant; *P < 0.05 and **P < 0.01.

We generated critical‐sized calvarial bone defects and used Gel@fMLP/SiO_2_‐FasL to investigate its effect on large bone defects and inflammation manipulation at the injury sites (**Figure** **4**A). 3D reconstruction images of micro‐computerized tomography (CT) analysis showed the appearance of new bone tissue in the Gel@fMLP/SiO_2_‐FasL group 8 w postoperation. However, little bone formation was observed in the Gel, Gel@SiO_2_, Gel@SiO_2_‐FasL and Gel@fMLP/SiO_2_ groups (Figure 4B). To quantify bone regeneration, the changes in bone volume to tissue volume (BV/TV) and bone surface to bone volume (BS/BV) within the calvarial defects were calculated. Semiquantitative analysis showed that the amount of bone regeneration was increased in the Gel@fMLP/SiO_2_‐FasL group and that there was almost no bone regeneration in the other groups (Figure 4C,D). Histological analysis showed that substantial cortical bone bridged the defect and integrated with the periphery after 8 w. However, only thin fibrous tissues were observed on and around the edge of the defect in the Gel, Gel@SiO_2_, Gel@SiO_2_‐FasL and Gel@fMLP/SiO_2_ groups (Figure 4E,F). Furthermore, a mature lamellar structure and bone marrow stromal areas were observed throughout the defect with continuous trabecular bone that extended over the defect. These data indicated that Gel@fMLP/SiO_2_‐FasL could significantly improve the repair of critical‐sized calvarial bone defects.

### 2.4 Gel@fMLP/SiO_2_‐FasL Induced a Transiently Heightened Inflammatory Response to Initiate Critical‐Sized Calvarial Bone Repair

**Figure 5:**
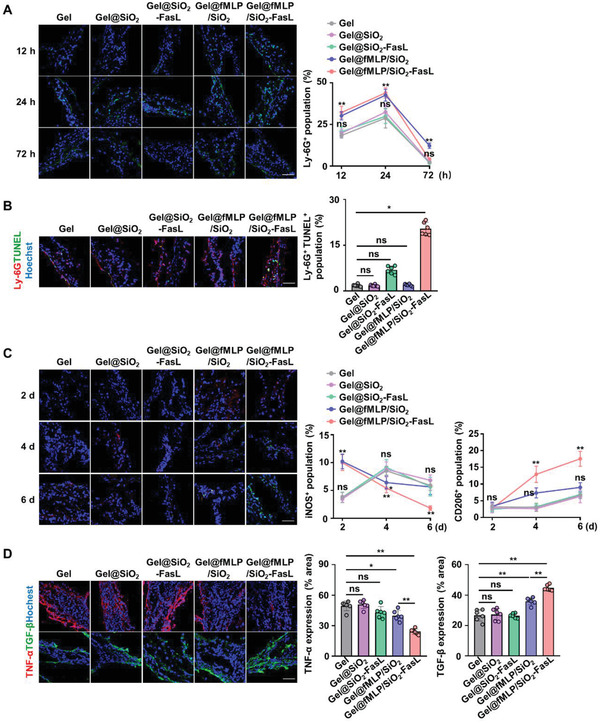
Inflammatory response manipulation by Gel@fMLP/SiO_2_‐FasL in critical‐sized calvarial bone defects. A) Representative fluorescence images and the percentage of neutrophils (Ly‐6G^+^ cells, green) that infiltrated the bone defects. Scale bars, 50 µm. B) Representative fluorescence images of apoptotic neutrophils (Ly‐6G^+^ TUNEL^+^ cells) in bone defects; Ly‐6G (red), TUNEL (green), and nuclei (blue). Scale bars, 50 µm. C) Representative fluorescence images of macrophage phenotypes in the bone defects. Scale bars, 50 µm. Quantitative analysis of iNOS^+^/CD206^+^ cells in each group. D) Representative fluorescence images of cytokine expression in bone defects. Scale bars, 50 µm. Quantitative analysis of TNF‐*α*/TGF‐*β* expression levels in each group. *n* = 6 per group. The data are presented as the means ± SD. In (A), (C), and (D), statistical analysis was performed using one‐way ANOVA. In (B), the CD206^+^ population of 4 d (in (C)) was analyzed using the Kruskal–Wallis *H*‐test. ns, not significant; *P < 0.05 and **P < 0.01.

To investigate whether the hybrid biomaterial could manipulate the inflammatory response during the repair of calvarial bone defects, the neutrophil infiltration level in the defect site was measured. Compared to those in the Gel, Gel@SiO_2_, and Gel@SiO_2_‐FasL groups, neutrophils were significantly increased in the Gel@fMLP/SiO_2_ and Gel@fMLP/SiO_2_‐FasL groups 12 h postoperation, and infiltration level peaked at 24 h, indicating the excellent capacity of the biomaterial to induce neutrophil chemotaxis (**Figure** **5**A). Moreover, the number of neutrophils decreased 72 h postoperation in all the groups and there was no significant difference between the Gel@fMLP/SiO_2_‐FasL group and the Gel group. However, there were more neutrophils in the Gel@fMLP/SiO_2_ group than in the other groups (Figure 5A). Moreover, neutrophil apoptosis in the defect sites was observed by Ly‐6G and terminal deoxynucleotidyl transferase dUTP nick end labeling (TUNEL) double‐positive staining. The immunofluorescence results showed that the apoptotic rate of neutrophils was significantly upregulated in the Gel@fMLP/SiO_2_‐FasL group, suggesting that FasL conjugation on the SiO_2_ promoted neutrophil apoptosis (Figure 5B).

In addition to neutrophils, macrophages in bone defects were also analyzed. As shown in the fluorescence images, proinflammatory macrophages (iNOS‐positive) were increased in the Gel@fMLP/SiO_2_ and Gel@fMLP/SiO_2_‐FasL groups 2 d postoperation compared to the Gel group, and these cells decreased over time, with the lowest number observed in the Gel@fMLP/SiO_2_‐FasL group. Proinflammatory macrophages peaked at 4 d in the Gel, Gel@SiO_2_, and Gel@SiO_2_‐FasL groups and decreased at 6 d (Figure 5C). In addition, the number of anti‐inflammatory macrophages increased over time in all groups, and more cells were observed in the Gel@fMLP/SiO_2_ and Gel@fMLP/SiO_2_‐FasL groups 4 d postoperation than in the other three groups. Anti‐inflammatory macrophages showed the greatest infiltration in the Gel@fMLP/SiO_2_‐FasL group at 6 d (Figure 5C). Finally, the expression of the proinflammatory cytokine TNF‐*α* and the anti‐inflammatory cytokine TGF‐*β* in bone defects was evaluated by immunofluorescence staining. The expression of TNF‐*α* decreased and TGF‐*β* increased in the Gel@fMLP/SiO_2_ and Gel@fMLP/SiO_2_‐FasL groups (Figure 5D), and the best efficacy was observed in the Gel@fMLP/SiO_2_‐FasL group, indicating the precise manipulation of the inflammatory process by the administration of Gel@fMLP/SiO_2_‐FasL. In general, these data indicated that Gel@fMLP/SiO_2_‐FasL could efficiently facilitate neutrophil recruitment and apoptosis and macrophage phenotypic transformation to initiate a transiently heightened inflammatory response in calvarial bone defects, benefiting bone repair.

### 2.5 Gel@fMLP/SiO_2_‐FasL Accelerated Wound Healing in Diabetic Cutaneous Defects

Injuries with systemic diseases, such as diabetic foot ulcers (DFUs), can result in chronic refractory wounds. Recently, a perturbed, ineffective inflammatory response was identified as a main contributor to the pathogenesis of DFUs, which was characterized by decreased neutrophil and macrophage recruitment and an overall poorly controlled inflammatory response.^[^ ^6^ ^]^ Therefore, further understanding of the inflammatory process in diabetic wounds would help in choosing efficient treatment options. First, we generated diabetic mice by an intraperitoneal injection of streptozotocin (STZ) to obtain increased fasting blood glucose and destroyed islets (Figure S6A,B, Supporting Information). Then, cutaneous defects were generated, and delayed healing of diabetic wounds was confirmed compared to that in wild‐type mice (Figure S6C, Supporting Information). Subsequently, the inflammatory responses in wild‐type and diabetic mice were evaluated by flow cytometry and immunofluorescence analysis. Neutrophils infiltrated in diabetic wounds more slowly with a lesser extent than that in wild‐type mice, in which neutrophils increased rapidly and peaked at 24 h postinjury (Figure S6D,E, Supporting Information). Proinflammatory macrophages (CD11b^+^Ly‐6C^high^iNOS^+^/iNOS^+^) in diabetic wounds increased slowly over time compared with those in wild‐type mice that rapidly peaked at 4 d postinjury (Figures S6F,H and S7, Supporting Information). Moreover, the number of anti‐inflammatory macrophages (CD11b^+^Ly‐6C^low^Arg‐1^+^/CD206^+^) increased gradually; however, there were fewer of these cells in the wounds of diabetic mice than in wild‐type mice (Figures S6G,H and S7, Supporting Information).

**Figure 6:**
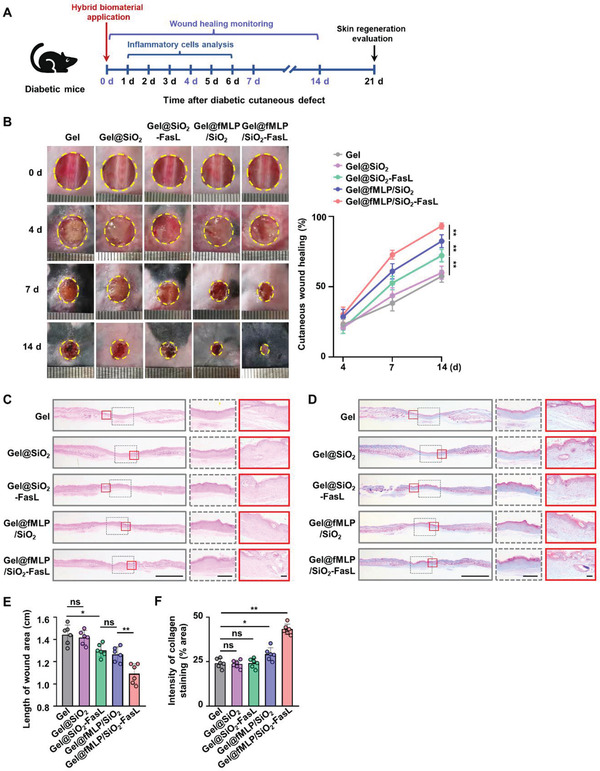
Gel@fMLP/SiO_2_‐FasL promotes the refractory wound healing of diabetic cutaneous defects. A) The experimental design of the hybrid biomaterial for the treatment of diabetic cutaneous defects. B) Representative photographs of the time course of diabetic cutaneous defects treated with hydrogel hybrids with different components. Quantitative analysis of the wound closure rate in each group. C) Representative images of H&E staining showing cutaneous defects. Scale bars, 1 mm (left), 2 mm (middle), 75 µm (right). D) Representative images of Masson staining showing cutaneous defects. Scale bars, 1 mm (left), 2 mm (middle), 75 µm (right). E) Quantitative analysis of the length of the wound area shown by H&E staining. F) Quantitative analysis of the collagen deposition level shown by Masson staining. *n* = 6 per group. The data are presented as the means ± SD. Statistical analysis was performed by one‐way ANOVA. ns, not significant; *P < 0.05 and **P < 0.01.

Accordingly, promoting sufficient neutrophil and macrophage recruitment and realigning the inflammatory process would provide a new strategy for refractory wound healing. To investigate the effect of the hybrid biomaterial on diabetic wounds, the hydrogels hybridized with different components were locally administered, and the wound area was monitored and measured at three different timepoints (**Figure** **6**A). The wound healing rate was elevated in the Gel@SiO‐FasL, Gel@fMLP/SiO_2_, and Gel@fMLP/SiO_2_‐FasL groups compared to the Gel@SiO_2_ and Gel groups, and the Gel@fMLP/SiO_2_‐FasL group had the fastest rate (Figure 6B). Then, H&E staining of the dissected skin tissues showed that Gel@fMLP/SiO_2_‐FasL significantly promoted wound healing compared to that in the other groups (Figure 6C), as demonstrated by a more integrated cutaneous structure with newly formed epithelium. In addition, the length of the wound area was greatly reduced after the administration of the different treatments, with the optimum effect in the Gel@fMLP/SiO_2_‐FasL group (Figure 6E). Moreover, better deposited and organized collagen was observed in the Gel@fMLP/SiO_2_‐FasL group than in the other groups, as shown by Masson staining (Figure 6D,F). These data demonstrated that Gel@fMLP/SiO_2_‐FasL could facilitate ineffective inflammatory response‐mediated chronic refractory wound healing.

### 2.6 Gel@fMLP/SiO_2_‐FasL Realigned the Transiently Heightened Inflammatory Response to Initiate Diabetic Wound Healing

**Figure 7:**
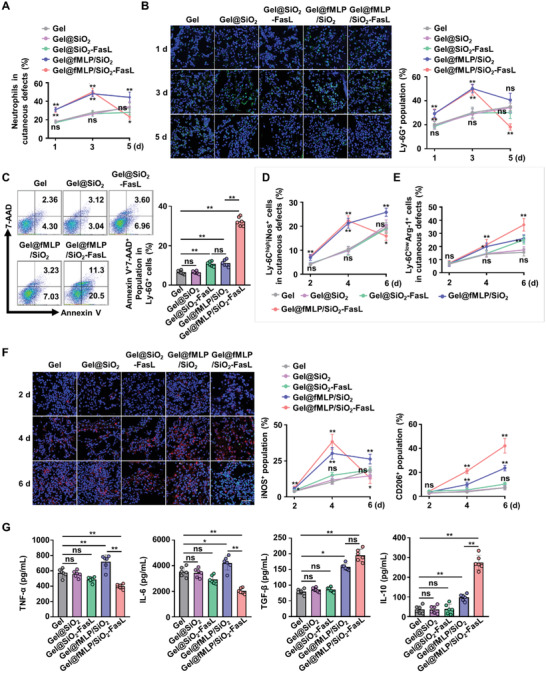
Inflammatory response realignment by Gel@fMLP/SiO_2_‐FasL in diabetic cutaneous wounds. A) Quantitative analysis of neutrophil infiltration in diabetic cutaneous defects was performed by flow cytometry. B) Representative fluorescence images and the percentage of neutrophils (Ly‐6G^+^ cells, green) in cutaneous defects. Scale bars, 50 µm. C) Representative flow cytometry plots and quantitative analysis of neutrophil apoptosis in cutaneous defects. D) Quantitative analysis of proinflammatory macrophages in cutaneous defects was performed by flow cytometry. E) Quantitative analysis of anti‐inflammatory macrophages in cutaneous defects was performed by flow cytometry. F) Representative fluorescence images of macrophage phenotypes in cutaneous defects. Scale bars, 50 µm. Quantitative analysis of iNOS^+^/CD206^+^ cells in each group. G) Cytokines expression in cutaneous defects was analyzed by ELISA. *n* = 6 per group. The data are presented as the means ± SD. In (A)–(G), statistical analysis was performed by one‐way ANOVA, except for TGF‐*β* expression (in (G)) which was analyzed by the Kruskal–Wallis *H*‐test. ns, not significant; *P < 0.05 and **P < 0.01.

To further explore the underlying mechanism of Gel@fMLP/SiO_2_‐FasL on diabetic cutaneous wound healing, the inflammatory cells infiltrated the defects were evaluated after the hybrid biomaterial was applied. The flow cytometry results showed that neutrophils were substantially increased in the Gel@fMLP/SiO_2_ and Gel@fMLP/SiO_2_‐FasL groups compared to the Gel group and peaked at 3 d postoperation, which significantly reversed the energy of inflammatory response in diabetic wounds (**Figure** **7**A and Figure S8A, Supporting Information). Moreover, the number of neutrophils declined dramatically at 5 d in the Gel@fMLP/SiO_2_‐FasL group compared to the Gel@fMLP/SiO_2_ group, indicating prompt apoptosis induced by FasL signaling. However, neutrophils in the Gel, Gel@SiO_2_ and Gel@SiO_2_‐FasL groups increased slowly over time, and these cells were decreased in the Gel@SiO_2_‐FasL group at 3 d compared to the Gel group (Figure 7A and Figure S8A, Supporting Information). Immunofluorescence analysis of Ly‐6G positive cells in the defects exhibited results that were similar to those of flow cytometry (Figure 7B). To verify the effect of FasL on neutrophils, neutrophil apoptosis in diabetic cutaneous defects was evaluated by flow cytometry (Figure S9, Supporting Information). The results showed that the apoptotic rates of Ly‐6G positive cells were elevated in the Gel@SiO_2_‐FasL and Gel@fMLP/SiO_2_‐FasL groups compared to the other groups and was higher in the Gel@fMLP/SiO_2_‐FasL group (Figure 7C).

Then, the macrophage distribution in the cutaneous defect was examined. The flow cytometry and immunofluorescence analysis results showed that the number of proinflammatory macrophages (Ly‐6C^high^iNOS^+^ cells/iNOS^+^ cells) was rapidly enhanced after Gel@fMLP/SiO_2_ and Gel@fMLP/SiO_2_‐FasL application, and decreased in the Gel@fMLP/SiO_2_‐FasL group after 4 d compared to that in the other groups (Figure 7D,F and Figure S8B, Supporting Information). In addition, anti‐inflammatory macrophages (Ly‐6C^low^Arg‐1^+^ cells/CD206^+^ cells) increased markedly in the defects, Gel@fMLP/SiO_2_‐FasL exerted the robust effect and Gel@fMLP/SiO_2_ resulted in a moderate effect on anti‐inflammatory macrophage infiltration (Figure 7E,F and Figure S8C, Supporting Information). Furthermore, the levels of multiple cytokines in cutaneous defects were examined by enzyme‐linked immunosorbent assay (ELISA). The results showed that the levels of proinflammatory factors (TNF‐*α* and IL‐6) were downregulated in the Gel@fMLP/SiO_2_‐FasL group, and the levels of anti‐inflammatory factors (TGF‐*β* and IL‐10) were distinctly elevated after Gel@fMLP/SiO_2_‐FasL treatment (Figure 7G).

Collectively, these data demonstrated that Gel@fMLP/SiO_2_‐FasL could promote neutrophil recruitment through fMLP release to initiate inflammation, and neutrophil apoptosis could be induced by FasL‐Fas interactions to trigger inflammation resolution in diabetic wounds, which subsequently helped to promote anti‐inflammatory macrophage transformation and upregulate anti‐inflammatory cytokines, realigning the inflammatory response to a transiently heightened process to achieve refractory wound healing.

## 3 Discussion

Many clues linking inflammation to damage repair and regeneration in mammals are conserved in lower organisms, suggesting that inflammation is an evolutionarily important process.^[^ ^3^ ^]^ Inflammatory responses immediately after injury mitigate infections, clear damaged cells and induce the recruitment, proliferation, and differentiation of mesenchymal stem cells to initiate the regenerative response, which serves as a scavenger of harmful substances and also a regenerative engine.^[^ ^27^, ^29^ ^]^ However, a prolonged and dysregulated inflammatory response can result in refractory wounds and even lead to many chronic and degenerative diseases.^[^ ^30^ ^]^ In healthy individuals with bone defects exceeding a critical size, inflammation stimulates the recruitment of stem cells and participates in the regulation of bone regeneration, but the void volume is larger than the self‐healing capacity of bone tissue, resulting in a nonhealing defect with a relatively chronic inflammatory state.^[^ ^5^, ^26^, ^27^ ^]^ In diabetic wounds, individuals with systemic immune disorders, an insufficient inflammatory response occurs at the beginning of the injury, and high levels of inflammation persist during the healing process, leading to a refractory wound.^[^ ^6^, ^7^ ^]^ Therefore, a timely and effective inflammatory response is one of the determinants of refractory wound healing. Moreover, a growing body of evidence has recently suggested that eliciting a transient acute inflammatory response could promote tissue regeneration.^[^ ^9^, ^31^ ^]^ However, precise manipulation of inflammatory process from initiation to resolution has not yet been achieved. In this study, we utilized a hybrid biomaterial to achieve orderly regulation of the recruitment and apoptosis of neutrophils and subsequently transformed the macrophage phenotype to gain a transiently heightened inflammatory response, which successfully facilitated the repair of critical‐sized calvarial bone defects and diabetic cutaneous wound healing.

Neutrophils are considered leader cells in host defense responses and respond to infection or tissue damage. Apart from participating in the proinflammatory response, neutrophils also pave the way for anti‐inflammatory and repair phases.^[^ ^16^, ^32^ ^]^ Insufficient or dysfunctional neutrophils due to disease or treatment side effects can lead to persistent chronic inflammation, delayed repair of tissue damage, and even the recurrence of serious infections, which are life‐threatening.^[^ ^33^, ^34^ ^]^ After injury, the migration of neutrophils is guided by diverse groups of extracellular molecular guidance cues. fMLP, a damage‐associated molecular pattern (DAMP) molecule generated by bacteria and damaged mitochondria, is considered a soluble end target chemoattractant, which means that neutrophils will preferentially migrate toward fMLP rather than other chemoattractants.^[^ ^35^, ^36^ ^]^ In this work, a pH‐responsive hydrogel was successfully designed with favorable machinery strength. Meanwhile, this hydrogel can carry exogenous fMLP and release it rapidly to induce neutrophil recruitment. After the first wave of neutrophils arrive at the site of injury, those cells produce other chemoattractants, such as leukotriene B4 (LTB4), which recruit more neutrophils to accomplish neutrophil swarming.^[^ ^37^, ^38^ ^]^ Moreover, chemokines secreted by neutrophils can also recruit proinflammatory monocytes and macrophages, in addition to the initial DAMP signals.^[^ ^10^ ^]^ Overall, locally elevated concentrations of fMLP could form a gradient that is favorable to neutrophil chemotaxis, which contributes to neutrophil migration and in turn promotes macrophage infiltration, thus initiating an effective inflammatory response.

After a successful response to acute injury, it is essential for neutrophils to be removed from the site either by apoptosis or reverse transmigration.^[^ ^11^ ^]^ However, neutrophils become activated, and their longevity increases by several fold during inflammation. If these cells are not removed in time, the persistence will result in disrepair and chronic inflammation.^[^ ^39^, ^40^ ^]^ Currently, the reverse migration of neutrophils remains controversial, and the exact mechanism(s), fate of reverse‐migrated neutrophils and occurrence of reverse migration in human disease are unclear.^[^ ^35^ ^]^ Apoptosis is generally considered a physiological form of cell death in neutrophils, including spontaneous apoptosis and death receptor‐induced apoptosis.^[^ ^22^ ^]^ The FasL/Fas system mediates typical apoptotic signaling in many cell types. Neutrophils possess a functional Fas receptor and are highly susceptible to Fas‐induced apoptosis with little Reactive Oxygen Species (ROS) production. However, only aggregated or membrane‐bound FasL can induce apoptosis.^[^ ^22^, ^41^, ^42^ ^]^ Therefore, we used SiO_2_ conjugated with FasL to induce apoptosis in active neutrophils. To accomplish the temporal regulation of this process, we used a pH‐responsive hydrogel to control the release of SiO_2_‐FasL. Activated neutrophils release MPO into the extracellular space generating hypohalous acids, particularly hypochlorous acid (HOCl). Thus, when many neutrophils arriving at the site of infection or inflammation, they create a low pH microenvironment during phagocytosis, degranulation or association with neutrophil extracellular traps (NETs).^[^ ^43^, ^44^ ^]^ Taking advantage of this characteristic, the pH‐responsive hydrogel dissociated, and SiO_2_‐FasL was released to induce apoptosis in activated neutrophils. In this study, neutrophils in the injury sites underwent apoptosis after the application of Gel@fMLP/SiO_2_‐FasL. Subsequently, apoptotic neutrophils were cleared by macrophages (efferocytosis). This clearance delivered signals that switched proinflammatory macrophages into anti‐inflammatory or so‐called proresolving/remodeling macrophages. The mediators produced by anti‐inflammatory macrophages counteract inflammatory signals and interact with mesenchymal stem cells to foster tissue repair and regeneration.^[^ ^45^, ^46^ ^]^ In this study, we found that Gel@fMLP/SiO_2_‐FasL successfully promoted macrophage phenotypic transformation and elevated the levels of anti‐inflammatory cytokines, which prepared for refractory wound healing. This process recruited abundant stem cells from bone sutures that further differentiated into functional cells for bone formation, completing the physiological process of intramembranous ossification and rebuilding the morphology and structure of calvarial bone.^[^ ^47^ ^]^ This treatment was also able to reprogram the microenvironment of diabetic cutaneous wounds to favor stem cell recruitment, which contributed to vascularization and re‐epithelization for wound healing.^[^ ^48^ ^]^

Overall, the hybrid biomaterial (Gel@fMLP/SiO_2_‐FasL) reprogrammed the initiation and resolution of inflammation by manipulating the recruitment and apoptosis of neutrophils and subsequent phenotypic transformation of macrophages in a timely and orderly manner. It induced a transiently heightened inflammatory response to fully mobilize the endogenous repair potential to promote tissue regeneration. Gel@fMLP/SiO_2_‐FasL facilitated the repair of the critical‐sized calvarial bone defects and diabetic cutaneous wound healing in mice, which provides a promising strategy for refractory wound therapy. However, large animal experiments will be needed to further evaluate the therapeutic effect, biological safety and feasibility of preclinical applications of the hybrid biomaterial, although no significant complications or death has been observed in mouse models. Thus, further studies will be required to optimize a system that is appropriate for different categories of refractory wounds.

## 4 Experimental Section

### Materials

CS (Mn = 20 kDa), rhodamine B, EDC, NHS and BSA (Mn = 66 kDa) were purchased from Sigma‐Aldrich. FPBA was purchased from Fisher Scientific. Sodium dihydrogen phosphate dihydrate (NaH_2_PO_4_·2H_2_O), sodium phosphate dibasic dodecahydrate (Na_2_HPO_4_·12H_2_O), dipotassium phosphate (K_2_HPO_4_), and sodium chloride (NaCl) were purchased from Macklin Inc. fMLP and FasL were purchased from Sigma‐Aldrich (F3506) and R&D Systems (6128‐SA/CF). Amino group‐modified SiO_2_ was purchased from XFNANO Materials Tech Co., Ltd (Nanjing, China). All the reagents were used as received.

### Preparation and Characterization of FPBA‐Modified CS

Well‐defined modified CS‐FPBA was prepared via the Schiff base reaction between amino groups and aldehyde groups. A coupling reaction between the aldehyde groups of FPBA and the amino group of CS was performed. Briefly, CS (2 g, ≈0.01 mmol CS) was dispersed in an acetic acid solution (1%, 200 mL). After 4 h of activation, FPBA (0.02 mmol) was slowly added to the mixed solution and stirred 12 h at room temperature, while a pH of 5.0 was maintained by the use of 1 m HCl. Unreacted FPBA was removed by a dialysis membrane tube with a molecular weight cutoff of 3.5 kDa in PBS (pH 5.0) for 48 h followed by dialysis against distilled water for 3 d. CS‐FPBA was obtained after lyophilization and was stored at 4 °C until use. CS was dispersed in an acetic acid solution, and CS‐FPBA were dispersed in distilled water, and their UV–vis spectra were measured by a WFZ‐26A UV–vis spectrophotometer (Tianjin Automatic Science Instrument Plant, China). To confirm conjugation, Fourier transform infrared spectroscopy (FT‐IR) was carried out using KBr disks by on a BIO‐RAD FT‐IR 3000 (BIO‐RAD Company, Hercules, USA), and the molar fractional ratio of conjugated carboxylic acid groups was calculated using the 1H NMR spectra of the products recorded by a Varian Inova‐500 M instrument (Varian Inc., Palo Alto, USA) with D2O as a solvent.

### Synthesis and Characterization of FasL‐Conjugated SiO_2_ (SiO_2_‐FasL)

FasL‐conjugated SiO_2_ (SiO_2_‐FasL) was synthesized by using a condensation reaction between the amino groups of SiO_2_ and carboxyl groups of FasL in the presence of EDC and NHS. Briefly, FasL (0.5%), EDC (0.01 mmol) and NHS (0.01 mmol) were dissolved in 2 mL of distilled water and activated for 2 h with stirring at 4 °C. Then SiO_2_ (200 µL) nanoparticles were added and stirred for 24 h at 4 °C, and the unbound FasL was removed by centrifugation. The resulting particles were collected, washed 3 times with distilled water and then freeze‐dried to obtain SiO_2_‐FasL. The morphology, size and zeta‐potential of SiO_2_‐NH_2_ and SiO_2_‐FasL were measured by TEM and laser particle size analysis. The samples for TEM observation were prepared by dropping 5 µL of solution onto the carbon‐coated copper grids, and their general surface morphologies were also observed by a Philips CM200 TEM equipped with an energy dispersive spectrometer. The loading percentage of FasL was determined by BCA analysis, with BSA representing FasL.

### Hydrogel Formation and Degradation

To prepare the hydrogel loaded with fMLP and SiO_2_‐FasL (Gel@fMLP/SiO_2_‐FasL), SiO_2_‐FasL complexes (10 µL) and 20 × 10^−6^ m fMLP were dispersed in 0.1 mL of PBS buffer solution (pH 7.4) containing 0.01 mg mL^−1^ of CS‐FPBA. Then the mixture was incubated at room temperature for 5 min to trigger the transformation from a nanoparticle suspension to a hydrogel via the free boric acid group at the terminus of FPBA with the cis‐o‐dihydroxyl group on the CS sugar unit to generate borate ester bonds and form the hydrogel of borate ester crosslinking. Hydrogel degradation tests were carried out by immersing the Gel@fMLP/SiO_2_‐FasL samples in PBS solutions (pH 5.5) at 37 °C.

### Two‐Stage fMLP and SiO_2_‐FasL Release Studies

To investigate two‐stage fMLP and SiO_2_‐FasL release, the hydrogel was formed as described above with modifications; FITC‐labeled SiO_2_‐BSA was used instead of pure SiO_2_‐FasL, and RhB was used instead of pure fMLP. For the first‐stage release analysis, the release profiles were examined in 0.1 m PBS at pH 7.4. The RhB‐loaded hydrogel was placed in 0.2 mL of PBS at 37 °C. At predetermined time intervals, the suspension was removed and replaced with fresh prewarmed PBS, and the suspensions were measured by fluorescence spectroscopy (excitation wavelength (EX) at 540 nm and emission wavelength (EM) at 625 nm for RhB) to determine the release of fMLP from the hydrogel into the buffer solution. For the second‐stage release analysis, the SiO_2_‐BSA‐FITC‐loaded hydrogel was placed in 2 mL of PBS (pH 6.8, pH 5.5) at 37 °C. The release profiles were examined at three different pH values (pH 7.4, pH 6.8, and pH 5.5). At predetermined time intervals, the suspension was removed and replaced with fresh prewarmed PBS. One milliliter of release medium was collected by centrifugation (500 r min^−1^ for 2 min at room temperature) and replaced with an equal amount of fresh PBS. The released percentage was determined by measuring the payload fluorescence using a fluorescence spectrophotometer (EX at 495 nm and EM at 520 nm for FITC) (Hitachi F‐7000).

### Mice

Eight week old female C57BL/6 mice were purchased from the Animal Center of Fourth Military Medical University (Xi'an, China). The mice were kept under specific pathogen‐free conditions with a 12 h light/12 h dark cycle. All mice had free access to food and water. All animal experiments were approved by the Institutional Animal Care and Use Committee of the Fourth Military Medical University (2020‐kq‐006).

### Primary Cell Culture

Murine neutrophils were isolated from the bone marrow of C57BL/6 mice using Histopaque separation media by a density gradient centrifugation.^[^ ^49^ ^]^ Briefly, bone marrow cells were harvested from the femurs and tibias, followed by erythrocyte lysis in Ammonium‐Chloride‐Potassium (ACK) lysis buffer (Beyotime, C3702). Then, the cell suspension was overlaid on top of the Histopaque 1077 (Sigma, 10771) layer with Histopaque 1119 (Sigma, 11191) in the bottom. After centrifugation, neutrophils at the interface of the Histopaque 1119 and Histopaque 1077 layers were collected. Then, the neutrophils were cultured in Roswell Park Memorial Institute (RPMI) 1640 (Gibco) supplemented with 10% fetal bovine serum (FBS; Invitrogen) and 1% penicillin/streptomycin (Invitrogen) or resuspended in Hanks balanced salt solution. Neutrophil purity was determined by flow cytometry (Beckman Coulter, USA) using allophycocyanin (APC)‐conjugated anti‐CD11b (BioLegend, 101212) and phycoerythrin (PE)‐conjugated anti‐Ly6G (BioLegend, 127607) antibodies.

Primary murine BMDMs were harvested from the bone marrow of C57BL/6 mice as previously described.^[^ ^50^ ^]^ The bone marrow was flushed from the femurs and tibias and then the red blood cells were lysed with ACK lysis buffer. After centrifugation, the cells were plated in Dulbecco's Modified Eagle Medium (DMEM) (high glucose) (Gibco) supplemented with 10% FBS, 1% penicillin/streptomycin and 20 ng mL^−1^ M‐CSF (Macrophage colony‐stimulating factor, CSF‐1) (PeproTech, 315–02) to induce cell maturation for at least 7 d for subsequent experiments. The induction of mature macrophages was evaluated by flow cytometry (Beckman Coulter, USA) using FITC‐conjugated anti‐CD11b (eBioscience, 11–0112) and PE‐conjugated anti‐F4/80 antibodies (Abcam, ab218761).

### Transwell Migration Assay

The hydrogel (Gel), hydrogel loaded with fMLP (Gel@fMLP), SiO_2_ (Gel@SiO_2_), and fMLP + SiO_2_ (Gel@fMLP + SiO_2_) were added to the lower chamber with 0.25% low free fatty acid BSA (MP Biomedicals, 0219989925) and incubated for 1 h. Hydrogel without chemoattractant (Gel) was used as a negative control. The supernatants were collected and added to a different lower chamber. Then, freshly isolated neutrophils were seeded to the upper chamber of a transwell plate (2 × 10^5^ well^−1^). After 45 min, the number of cells that had migrated into the lower chamber was determined by flow cytometry. For fluorescence analysis, SiO_2_ labeled with FITC was loaded in the lower chamber in the Gel + SiO_2_ and Gel@fMLP + SiO_2_ groups in the presence of the supernatants of hydrogels prepared with/without fMLP. Neutrophils were labeled with PKH26 (Sigma‐Aldrich, MINI26) and added to the upper chamber. After 45 min, the plates were observed under a fluorescence microscope and the number of PKH26‐labeled neutrophils in the lower chamber was quantified.

### Apoptosis Assays

Freshly isolated neutrophils were cultured in RPMI 1640 supplemented with 10 × 10^−6^ m fMLP. After 1 h, the neutrophils were activated. Then, the activated neutrophils were cultured with SiO_2_ or SiO_2_ conjugated with FasL (SiO_2_‐FasL). Single‐cultured neutrophils were used as a control. After 6 h, the cells were collected for subsequent use. Apoptotic neutrophils were examined by a PE Annexin V Apoptosis Detection Kit I (BD Biosciences, 559763) and analyzed by flow cytometry. Briefly, the cells were washed with cold PBS and resuspended in 100 µL of binding buffer. PE Annexin V and 7‐amino‐Actinomycin D (7‐AAD) solution (5 µL each) were added and incubated for 15 min. Another 400 µL of binding buffer was added, and then the samples were analyzed with a flow cytometer within 1 h. The apoptotic rate of neutrophils is the sum of Annexin V^+^/7‐AAD^−^ cells and Annexin V^+^/7‐AAD^+^ cells.

### Neutrophil and Macrophage Coculture

Mature macrophages were stimulated with LPS (1 µg mL^−1^) (MedChemExpress, HY‐D1056) to induce inflammation. Moreover, PBS, activated neutrophils (neutrophils), activated neutrophils cultured with SiO_2_ (Neu + SiO_2_) and activated neutrophils cultured with SiO‐FasL (Neu + SiO_2_‐FasL) were added to the medium. Unstimulated macrophages were used as controls. After 24 h, the supernatants and macrophages were collected for subsequent analyses.

### Western Blot Analysis

Cells were lysed with radio‐immunoprecipitation assay (RIPA) buffer (Beyotime, China) containing protease inhibitor (Roche, 04693132001) on ice. Protein quantification was performed with BCA protein assay reagent (Beyotime). Twenty micrograms of protein were separated by sodium dodecyl sulfate (SDS) polyacrylamide gel electrophoresis and transferred onto polyvinylidene fluoride (PVDF) membranes (Millipore, Germany). After being blocked in 5% BSA (MP Biomedicals) for 1 h at room temperature, the membranes were incubated at 4 °C overnight with primary antibodies against Fas (Santa Cruz Biotechnology, sc‐1023), MPO (Abcam, ab208670), Bcl‐2 (Cell Signaling, 3498S), Bax (Cell Signaling, 2772), Caspase‐3 (Cell Signaling Technology, 9662S), BID (Cell Signaling Technology, 2003S), glyceraldehyde‐3‐phosphate dehydrogenase (GAPDH) (CWBIO, CW0100), iNOS (Cell Signaling Technology, 13120S), mannose receptor (Abcam, ab125028), Arginase‐1 (Cell Signaling Technology, 93668S), and *β*‐actin (CWBIO, CW0096). The membranes were washed with tris buffered saline tween (TBST) and incubated with appropriate secondary antibodies for 1 h at room temperature. Finally, the blots were visualized with a Western‐Light Chemiluminescent Detection System (Tanon‐Bio, China).

### Diabetic Cutaneous Wound Healing Model

Mice were intraperitoneally injected with a freshly prepared solution of STZ (MP Biomedicals, 02100557C) (50 mg kg^−1^, dissolved in citrate buffer, pH 4.5). For 5 consecutive days, after the mice underwent a 12 h fast for the first day and a 5 h fast for the next 4 d. Fasting blood glucose levels were measured 7 d after the last injection of STZ using a glucometer (Roche, Switzerland). Mice were considered diabetic when blood glucose levels exceeded 11.1 mmol L^−1^ for 2 consecutive days.

Mice were anesthetized by an intraperitoneal injection of pentobarbitone sodium (40 mg kg^−1^). After being shaved and cleaned, a 10‐mm circular, full‐thickness dorsal wound was aseptically generated. The mice were randomly assigned to five groups (n = 6 in each group) and hydrogel (Gel), hydrogel hybrid with SiO_2_ (Gel@SiO_2_), hydrogel hybrid with SiO_2_ conjugated with FasL (Gel@SiO_2_‐FasL), hydrogel hybrid with fMLP and SiO_2_ (Gel@fMLP/SiO_2_), hydrogel hybrid with fMLP and SiO_2_‐FasL (Gel@fMLP/SiO_2_‐FasL) were locally applied. After the operation, the wound was covered by surgical dressings (3 M, 9546) and removed uniformly. The wound sizes were photographed and measured with Image J at the indicated time points (days 0, 4, 7, and 14). The mice were sacrificed 1, 2, 3, 4, 5, 6, and 21 d postoperation and the injured areas were collected for further analysis.

### Critical‐Sized Calvarial Bone Defect Model

The mice were anesthetized, and a round, 3.5 mm diameter defect centered on the parietal calvarial bone was then manually made using a sterile scalpel. The calvarial disk was removed carefully without disturbing the underlying dura mater. After careful hemostasis, hydrogel (Gel), hydrogel hybrid with SiO_2_ (Gel@SiO_2_), hydrogel hybrid with SiO_2_ conjugated with FasL (Gel@SiO_2_‐FasL), hydrogel hybrid with fMLP and SiO_2_ (Gel@fMLP/SiO_2_), and hydrogel hybrid with fMLP and SiO_2_‐FasL (Gel@fMLP/SiO_2_‐FasL) (*n* = 6 in each group) were placed onto the defects. Finally, the incisions were closed with 4‐0 Nylon sutures and the mice were monitored appropriately. The mice were sacrificed 12 h, 1 d, 2 d, 3 d, 4 d, 6 d, and 8 weeks postoperation, and calvarial bone was collected for further analysis.

### Dermal Inflammatory Cell Analysis by Flow Cytometry

Mice were anesthetized and the injured skin was collected. After being washed by cold PBS, the skin was digested with 2 mg mL^−1^ Dispase I (Sigma‐Aldrich, D4818) for 30 min at 37 °C. Then the epidermis was manually removed and the dermis was transferred to a clean culture plate, followed by incubation with 1 mg mL^−1^ collagenase I (Gibco, 17018029) for 1 h. An equal volume of RPMI 1640 supplemented with 10% FBS was added, and the supernatant was collected. After being filtered through a 100 µm cell strainer, a single‐cell suspension was generated. The erythrocytes were lysed with ACK lysis buffer, and the cells were harvested. To analyze surface markers, cells were stained with FITC‐conjugated anti‐CD11b (BioLegend, 101206), PE‐conjugated anti‐Ly‐6G (BioLegend, 127607) and FITC‐conjugated anti‐Ly‐6C antibodies (BioLegend, 128005) for 30 min on ice in the dark. For intracellular staining, fixation and permeabilization were performed with an Intracellular Fixation & Permeabilization Buffer Set (Invitrogen, 88–8824–00) at room temperature for 1 h. Then, the cells were stained with PE‐conjugated anti‐iNOS (BioLegend, 696806) or PE‐conjugated anti‐Arg‐1 antibodies (Invitrogen, 12‐3697‐82) for 1 h. The cells were washed twice and acquired on a flow cytometer (Cytomics FC 500, Beckman‐Coulter). The percentages of positive cells were analyzed using FlowJo_V10 software.

### Histological Analysis

The calvarial bone was decalcified with 17% ethylene diamine tetraacetic acid (MP Biomedicals, 10378‐22‐0) for 4 d. The decalcified calvarial bone, skin and pancreas were then embedded in paraffin and 4 µm thick sections were obtained. The sections were deparaffinized and stained with H&E and Masson according to the manufacturer's instructions. The slides were observed under a light microscope and images were obtained using a stereomicroscope (Leica, Germany).

### Immunofluorescence Analysis

The glass coverslips of macrophage and neutrophil coculture system were fixed with 4% paraformaldehyde at room temperature for 15 min. The decalcified calvarial bone and the skin were embedded in the optimal temperature compound (Leica, United States), and 10 µm thick sections were obtained. The coverslips and the sections were permeabilized with 0.1% Triton X‐100 (Sigma‐Aldrich, 93443) for 10 min and subsequently blocked using 5% BSA at 37 °C for 1 h. For neutrophil analysis, the sections were incubated with anti‐Ly‐6G primary antibody (Abcam, ab25377) overnight at 4 °C and then incubated with the FITC‐conjugated anti‐rat secondary antibody (Jackson ImmunoResearch, 112‐095‐003) at room temperature for 1 h. For macrophage analysis, the coverslips and the sections were incubated with Alexa Fluor 488‐anti‐mannose receptor (Abcam, ab195191) and Alexa Fluor 647‐anti‐iNOS (Abcam, ab209027) antibodies overnight at 4 °C. Nuclear counterstaining was performed with Hoechst 33342 (Sigma‐Aldrich, 14533) at room temperature for 10 min. Images were captured using a confocal microscope (Nikon, Japan).

### Micro‐CT Analysis

For calvarial bone‐defect repair examination, high‐resolution micro‐CT analyses were performed using explore Locus SP Pre‐Clinical Specimen microcomputed tomography (GE Healthcare, USA). The samples were fixed in a cylindrical holder with the coronal section of the calvarial bone in the horizontal position. Then the samples were scanned with the direction parallel to the coronal section. 3D histomorphometric analysis were performed using software provided by a desktop micro‐CT system (GE Healthcare, USA). BV/TV and SA/BV were quantitatively analyzed.

### ELISA

Fresh skin wound samples were harvested and homogenized in RIPA buffer containing a protease inhibitor on ice. Then, the supernatants were collected after centrifugation at 12 000 rpm for 10 min at 4 °C. Supernatant protein levels in skin wound samples and macrophage‐neutrophil coculture systems were determined using mouse TNF‐*α*, IL‐6, IL‐10, and TGF‐*β* ELISA kits (MultiSciences, China). All procedures were performed according to the manufacturer's instructions.

### Statistical Analysis

All data are expressed as the means ± standard deviation (SD). Statistical comparisons between datasets were performed by analyzing normality and variance. Statistical significance between two groups was calculated by Student's *t*‐tests (two‐tailed). One‐way analysis of variance (ANOVA) was used to compare differences among multiple groups, and Tukey's post hoc test was used for multiple post hoc comparisons to determine the significance of differences between the groups after one‐way ANOVA. The Kruskal–Wallis *H*‐test or Mann–Whitney *U*‐test was used if the data did not follow a normal distribution. *P* values less than 0.05 were considered statistically significant. Statistical analysis was performed using SPSS software (IBM, 19.0).

## Conflict of Interest

The authors declare no conflict of interest.

## Author Contributions

X.L., G.D., and Z.L. contributed equally to this work. X.L., G. D., and Z. L. contributed to the study design, data collection, and paper preparation. X.W. and R.J. contributed to the hybrid biomaterial preparation. Y.L., H.K., X.H., X.Y., X.Y., and S.L. contributed to the in vitro experiments. M.W., H.G., F.D., and H.X. contributed to the animal study. S.L., Y.J., and K.X. developed the concept, supervised the research, and critically revised the paper. All authors contributed to the paper and approved the final paper.

## Supporting information

Supporting Information

Supplemental Table 1

## Data Availability

The data that support the findings of this study are available from the corresponding author upon reasonable request.
