# Supplementary material for: Hybrid Biomaterial Initiates Refractory Wound Healing via Inducing Transiently Heightened Inflammatory Responses
Source: Adv Sci (Weinh). 2022 May 23;9(21):2105650. doi: 10.1002/advs.202105650 (PMC9313498; doi:10.1002/advs.202105650)
Supplement: Supplementary file 1 — Supporting Information [file ADVS-9-2105650-s001.pdf]

Supporting Information

Hybrid Biomaterial Initiates Refractory Wound Healing *via* Inducing Transiently Heightened Inflammatory Responses

Xuemei Liu, Geng Dou, Zihan Li, Xiangdong Wang, Ronghua Jin, Yao Liu, Huijuan Kuang, Xiaoyao Huang, Xiaoxue Yang, Xiaoshan Yang, Siying Liu, Meiling Wu, Hao Guo, Feng Ding, Haokun Xu, Shiyu Liu, Yan Jin*, and Kun Xuan**

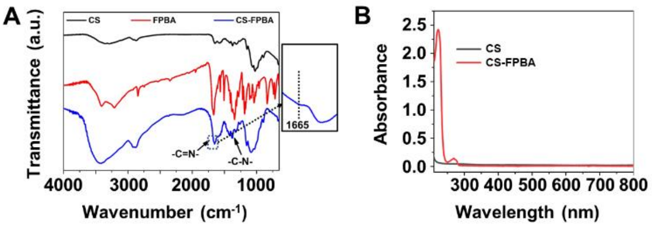

Figure S1. Conjugation of FPBA to CS. A. IR spectrometry of CS, FPBA, and CS-FPBA. B. UV spectra of CS-FPBA.

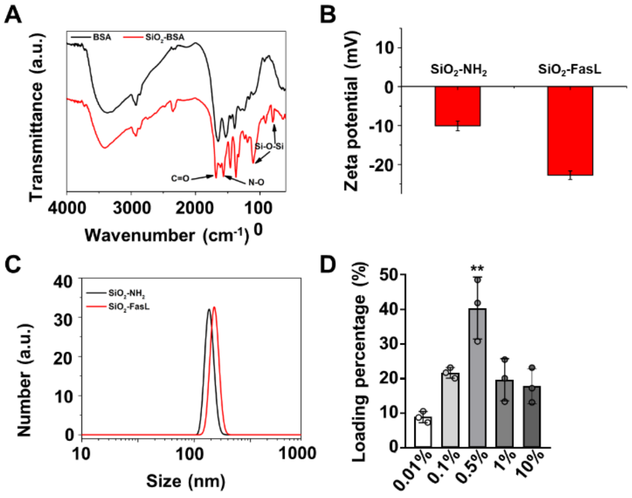

Figure S2. FasL immobilized on SiO_2. A. IR spectrometry of BSA and SiO_2-BSA (representing SiO_2-FasL). B. The zeta potentials of SiO_2-NH_2 and SiO_2- FasL (pH 7.4). C. The size distributions of SiO_2-NH_2 and SiO_2-FasL. D. The loading percentage of FasL (BSA representing FasL). Statistical analysis was performed by using one-way ANOVA. $**P < 0.01$.

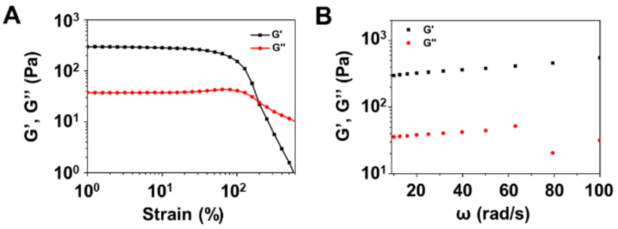

Figure S3. Rheological analysis of Gel@fMLP/SiO_2-FasL in frequency sweep mode.

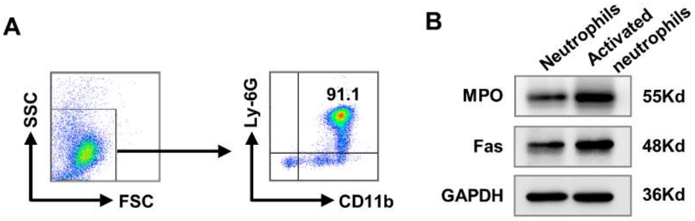

Figure S4. Characterization and activation of primary isolated neutrophils. A) Representative flow cytometry plots of neutrophils isolated from mouse bone marrow. B) Western blot analysis of MPO and Fas expression in neutrophils before and after activation with 10 μM fMLP for 1 h. The experiments were repeated three times independently to confirm the results.

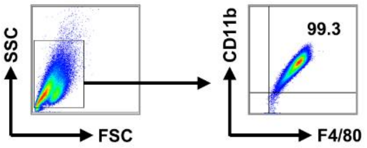

Figure S5. Characterization of primary isolated macrophages. Representative flow cytometry plots of macrophages isolated from mouse bone marrow. The experiments were repeated three times independently to confirm the results.

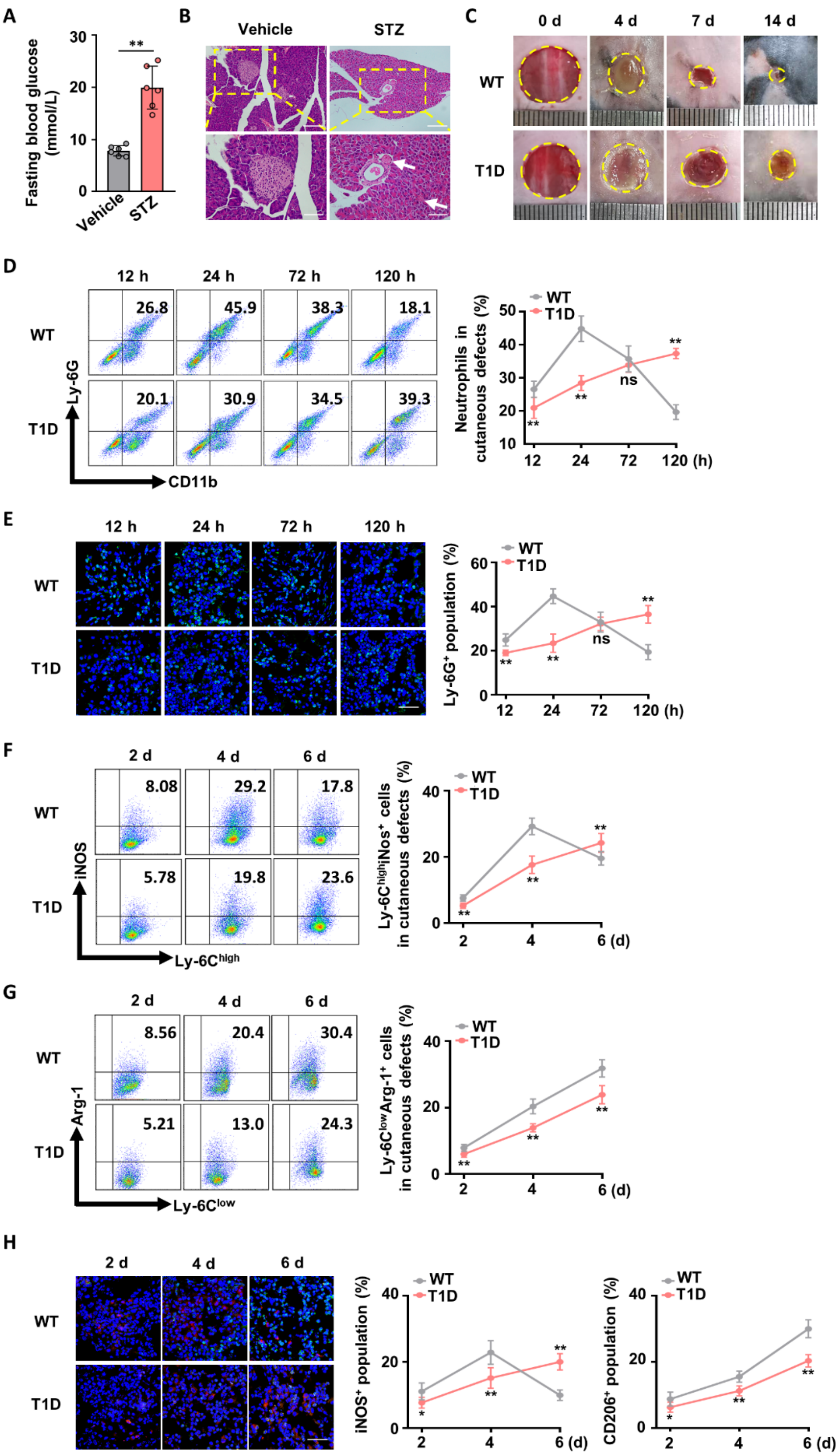

Figure S6. Inflammatory cells infiltrated the full-thickness cutaneous defects of healthy mice and diabetic mice. A) Fasting blood glucose of mice induced by STZ injection (STZ group) compared with mice injected with sodium citrate (Vehicle group). B) Representative images showing H&E staining of the pancreas of mice induced by STZ injection compared with mice

injected with sodium citrate. The white arrowhead indicates the destroyed islets. Scale bars, 100 μm (top), 50 μm (bottom). C) Representative photographs of cutaneous wound healing in healthy (WT) and diabetic mice (T1D) over time. D) Representative flow cytometry plots and quantitative analysis of neutrophils in the cutaneous defects of healthy and diabetic mice. E) Representative fluorescence images and quantitative analysis of neutrophils (Ly-6G$^+$ cells) in cutaneous defects. Scale bars, 50 μm. F) Representative flow cytometry plots and quantitative analysis of proinflammatory macrophages in the cutaneous defects of healthy and diabetic mice. G) Representative flow cytometry plots and quantitative analysis of anti-inflammatory macrophages in the cutaneous defects of healthy and diabetic mice. H) Representative fluorescence images of macrophages and quantitative analysis of the percentage of proinflammatory macrophages (iNOS$^+$ cells) /anti-inflammatory macrophages (CD206$^+$ cells) in the cutaneous defects of healthy and diabetic mice. Scale bars, 50 μm. n = 6 per group. The data are presented as the means ± SD. In A, statistical analysis was performed by using the Mann–Whitney U test. In D-H, statistical analysis was performed by using Student's t test (two-tailed). ns, not significant; *P < 0.05 and **P < 0.01.

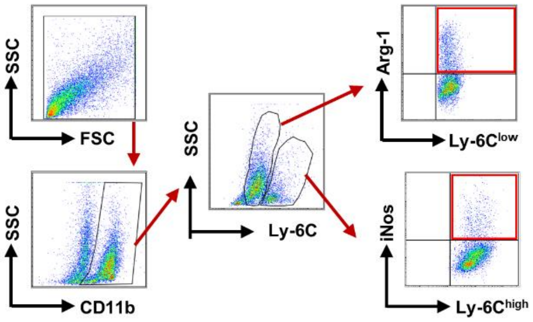

Figure S7. Gating strategy of macrophages in diabetic cutaneous wounds.

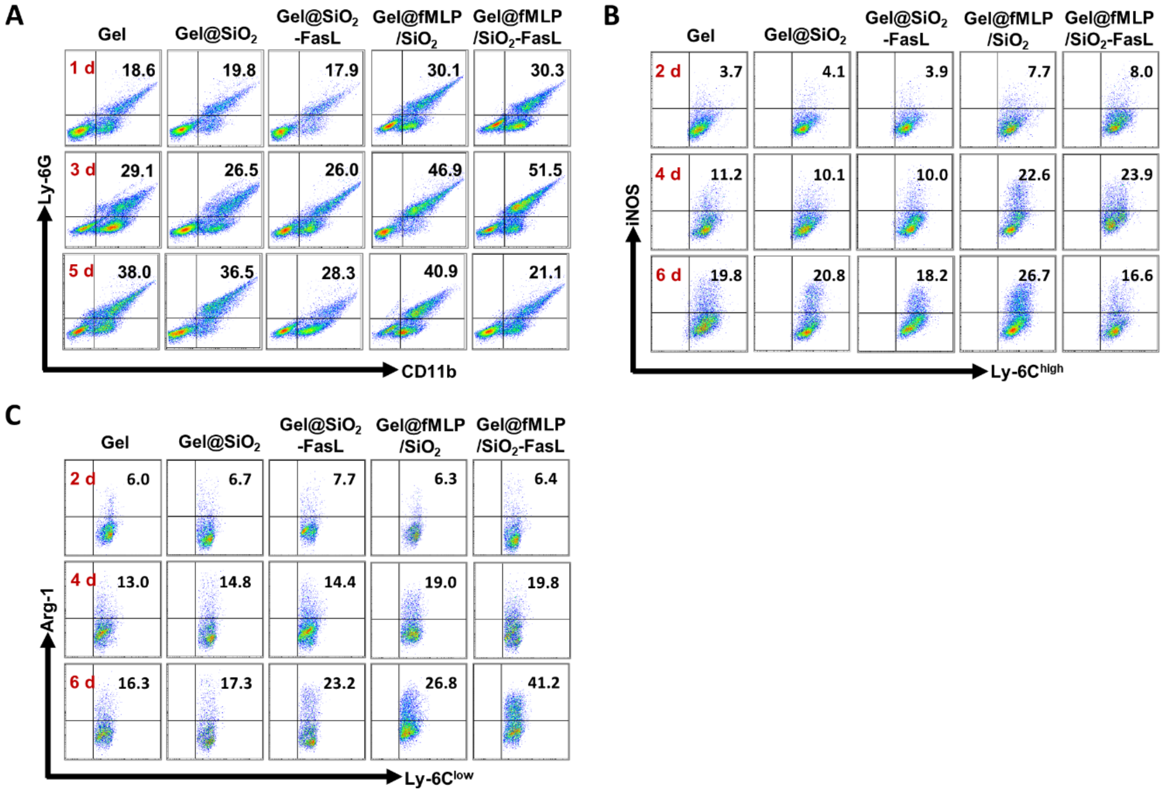

Figure S8. Representative flow cytometry plots of neutrophils and macrophages in diabetic cutaneous wounds after different treatments. A) Representative flow cytometry plots of neutrophils that infiltrated wounds treated with hydrogels hybridized with different components. B) Representative flow cytometry plots of proinflammatory macrophages that infiltrated wounds treated with hydrogels hybridized with different components. C) Representative flow cytometry plots of anti-inflammatory macrophages that infiltrated in the wounds treated with hydrogels hybridized with different components.

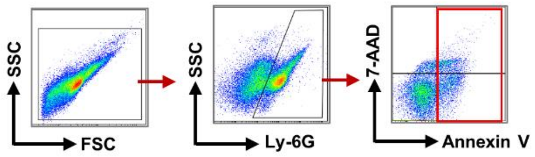

Figure S9. Gating strategy of apoptotic neutrophils in diabetic cutaneous wounds.

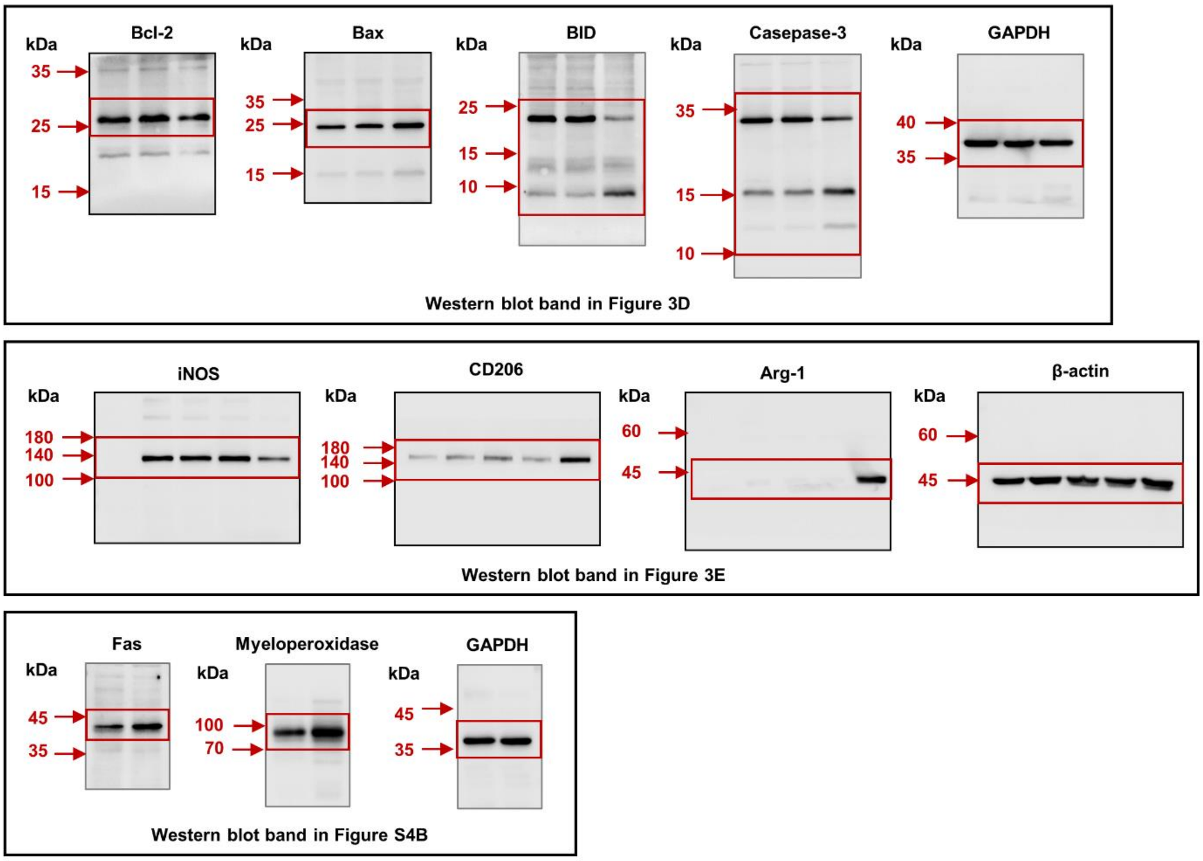

Figure S10. Uncropped images showing the Western blot bands in this study.
